# Changes of body composition after valve surgery in patients with mitral valve disease

**DOI:** 10.1371/journal.pone.0203798

**Published:** 2018-09-21

**Authors:** Sung-Ai Kim, Min-Kyung Kang, Chi Young Shim, Sak Lee, Byung-Chul Chang, Jong-Won Ha

**Affiliations:** 1 Division of Cardiology, Hallym Sacred Heart Hospital, Hallym University College of Medicine, Anyang, Korea; 2 Division of Cardiology, Severance Cardiovascular Hospital, Yonsei University College of Medicine, Seoul, Korea; 3 Division of Cardiovascular Surgery, Severance Cardiovascular Hospital, Department of Thoracic and Cardiovascular Surgery, Yonsei University College of Medicine, Seoul, Korea; University of Liège, BELGIUM

## Abstract

**Background:**

Patients with chronic heart failure have alteration in body composition as a reduction in fat mass, lean body mass and bone mass. However, body wasting in valvular heart disease and the impact of corrective valvular surgery on body composition has not been investigated.

**Objectives:**

We hypothesized that body wasting in severe mitral valve (MV) diseases is reversible through MV surgery.

**Methods:**

Forty eight patients who were scheduled to undergo MV surgery were consecutively enrolled after excluding patients with combined valvular heart disease, ischemic heart disease, cardiomyopathies, and diseases or who were taking medications that could affect metabolism. All patients were subjected to simplified nutritional assessment questionnaire (SNAQ) for appetite, laboratory tests, echocardiography, and dual-energy X-ray absorptiometry (DXA) before and one year after MV surgery.

**Results:**

One year after MV surgery, the patients showed increased appetite and improved laboratory data as well as hemodynamic improvement.When we classified the patients according to the primary MV lesion, no changes in body weight were observed in both patients with mitral regurgitation (MR) and mitral stenosis (MS). However, significant increase in bone mineral density and body fat percentage were observed in patients with MR and not in patients with MS. In patients with MR, patients with Δfat ≥ 2% showed significantly higher pre-operative estimated right ventricular systolic pressure (eRVSP) level and greater decrease in eRVSP after surgery than those with Δfat < 2% and both ΔSNAQ and Δfat showed significant negative relationship with ΔeRVSP, respectively.

**Conclusions:**

In patients with severe MV disease, corrective MV surgery led to favorable outcomes in wasting process as well as hemodynamic improvement. Particularly, right ventricular pressure overload showed a close association with the changes in appetite and body fat percentage in patients with MR.

## Introduction

Wasting process in heart failure (HF) has been known as an independent risk factor for mortality [1]. Although the mechanism of apparent body wasting in HF is not yet clarified, increased catabolism and decreased anabolism by dietary deficiency, malabsorption, and metabolic dysfunction have been proposed to be responsible for the development of cardiac cachexia [2–3]. Consequently, body composition of cachectic patients has a reduction in fat mass, lean body mass and bone mass [4–5], and the wasting process has significant influence on the quality of life as they experience easy fatigue and muscle weakness as well as poor prognosis.

Especially, body wasting in valvular heart diseases has received little attention. A previous report has shown that all patients with severe mitral valve (MV) disease who underwent MV surgery (replacement/annuloplasty) had experienced a marked increase in appetite, postoperatively [6]. However, the effect of corrective valve surgery on nutritional status and body composition is not clearly understood.

In this study, we enrolled patients with severe MV disease who underwent MV surgery, and analyzed their body composition by dual-energy X-ray absorptiometry (DXA) and appetite using questionnaire, as well as anthropometric and laboratory parameters, before and one year after MV surgery. We hypothesized that the wasting process in patients with severe MV disease is reversible through corrective MV surgery.

## Materials and methods

### 1. Study population

We prospectively enrolled 48 patients with severe mitral valvular disease scheduled for MV surgery (repair or replacement). Patients with combined significant valvular heart disease, ischemic heart disease, cardiomyopathies, right ventricular (RV) dysfunction, previous MV surgery, diabetes, cirrhosis or active hepatitis, thyroid disease, chronic inflammatory or infectious diseases, and active malignancy (i.e. diseases that could affect metabolism, nutritional status and body composition), known pregnancy, use of steroid, antidepressant, and other appetite stimulants were excluded.

This study was approved by the Ethics Committee and Institutional Review Board of Yonsei University College of Medicine, and written informed consent was obtained from all patients prior to enrolment.

### 2. Short nutrition assessment questionnaire (SNAQ)

Appetite was measured using the Simplified Nutritional Appetite Questionnaire (SNAQ) [7]. The SNAQ includes four questions based on following numerical scale: A = 1, B = 2, C = 3, D = 4, E = 5.

1. My appetite is (A. very poor/ B. poor/ C. average/ D. good/ E. very good)
2. When I eat (A. I feel full after eating only a few mouthfuls/ B. I feel full after eating about a third of a meal/ C. I feel full after eating over half of meal/ D. I feel full after eating most of the meal/ E. I hardly ever feel full)
3. Food tastes (A. Very bad/ B. Bad/ C. Average/ D. Good/ E. very good)
4. Normally I eat (A. Less than one meal a day/ B. One meal a day/ C.Two meals a day/ D.Three meals a day/ E. More than 3 meals a day).

The total SNAQ score range from four to 20. Those with SNAQ scores <14 has been reported to be at significant risk for weight loss >5% within 6 months, with a sensitivity of 81.3%, and a specificity of 76.4% [7].

### 3. Echocardiography

Echocardiography was performed using a GE Vingmed System 7 ultrasound system (Horten, Norway) with a 2.5-MHz transducer for image acquisition. Standard two-dimensional measurements were obtained with the patient in the left lateral position. Left ventricular ejection fraction (LVEF) was measured using the modified Simpson method. Left ventricular (LV) outflow tract diameter (LVOTd) was measured in the parasternal long axis view in early systole immediately adjacent to the point of aortic cusp insertion. LVOT velocity–time integral (VTI_LVOT_) measured using pulsed wave Doppler was recorded from the apical five chamber view in the LVOT, by placing the sample volume approximately five mm apically from the aortic valve and aligning it parallel with blood flow. LV stroke volume (SV) was obtained by multiplying the LVOT area by VTI_LVOT_ using the formula SV (ml) = LVOT area (cm^2^) x VTI_LVOT_ (cm). Left atrial (LA) volumes were calculated by biplane Simpson method using apical four and two chamber views. The LA volume index (LAVI) was calculated as LA volume/body surface area (mL/m^2^). Right ventricular systolic pressure (RVSP) was estimated from the maximal tricuspid regurgitant jet velocity by using the modified Bernoulli equation (4V^2^) and the addition of estimated right atrial pressure on the basis of inferior vena cava diameter and collapsibility as previously described [8].

Echocardiographic severity of valvular stenosis or regurgitation was assessed according to EAE/ASE recommendations for clinical practice [9–10].

### 4. Dual-energy X-ray absorptiometry (DXA)

Body compositions were measured by a standardized method, using DXA and assessed using whole body composition software (BHR-140-P Discovery A, Hologic Inc., Bedford, MA, USA). All scans were acquired and analyzed according to each manufacturer's standard scanning and positioning protocols. DXA scanned the weight (g) of total mass, fat mass, lean body mass, and the percentage of fat (fat mass divided by total mass) was calculated. Bone mineral content was expressed in g, and bone mineral density (BMD) in g/cm^2^. The coefficient of variation for both spine and whole body calibration phantoms was less than 1%.

### Statistical analysis

Continuous variables are represented as mean (± standard deviation) or median (inter-quartile range) if non-normally distributed. Categorical variables are presented as counts and proportions. Comparisons between before and after MV surgery were performed with paired t-test or Wilcoxon signed rank test for paired continuous data. Man-Whitney U test was used for non-parametric comparisons of continuous data between groups. Correlations of pre-operative SNAQ and ΔSNAQ with other variables were performed using Pearson’s coefficient (r) or Spearman correlation. The significance level was set at p <0.05. Statistical analyses were performed using SPSS version 23.0 (IBM, Armonk, NY, USA).

## Results

The mean age of the study population was 52 ± 13 years, and 24 (50%) patients were male.

Mean EUROSCORE II was 1.52 ± 1.46%. The reasons for MV surgery were mitral regurgitation (MR) in 32 (67%) patients and mitral stenosis (MS) in 16 (33%) patients.

In patients with MR, MV repair was performed in 26 (81%) and prosthetic MV replacement in 6 (19%) patients. All the patients with MS underwent MV replacement.

We classified the patients according to the primary MV lesions (MR vs MS) and Table 1 shows the changes in anthropometric, echocardiographic, laboratory measurements and body composition before and after MV surgery.

**Table 1:** Changes in anthropometric, echocardiographic, laboratory measurements and body composition before and after mitral valvular surgery according to the primary valve lesion.

Variable	Before surgery			1 year after surgery		
	All	MR	MS	All	MR	MS
	(n = 48)	(n = 32)	(n = 16)	(n = 48)	(n = 32)	(n = 16)
Sytolic BP, mmHg	115 ± 12	115 ± 11	111 ± 12	123 ± 14**	124 ± 13	121 ± 15
Diastolic BP, mmHg	71 ± 10	71 ± 10	71 ± 11	78 ± 11**	77 ± 12	82 ± 10
Heart rate, bpm	72 ± 10	72 ± 11	72 ± 10	76 ± 9	77 ± 8	75 ± 11
Body weight, kg	62 ± 12	64 ± 13	54 ± 9†	62 ± 13	65 ± 13	56 ± 9
Height, cm	164 ±9	165 ± 11	159 ± 9†	164 ±9	166 ± 10	160 ± 8
BMI, kg/m^2^	22.6 ± 3.1	23.3 ± 3.1	21.5 ± 2.3†	22.8 ± 3.2	23.6 ± 3.4	21.4 ± 2.2
SNAQ score	13.5 ± 2.1	13.7 ± 2.0	12.8 ± 2.4	15.1 ± 1.8**	15.4 ± 1.6**	14.4 ± 1.9*
LVEF, %	64 ± 9	68 ± 5	58 ± 11‡	63 ± 7	63 ± 8**	62 ± 5
Stroke volume, ml	56.1 ± 14.4	60 ± 12	48 ± 12†	67.3 ± 12.4**	69 ± 11**	60 ± 10*
LA volume index, ml/m^2^	78.3 ± 51.3	66 ± 41	102 ± 62†	45.4 ± 30.3**	41 ± 29**	53 ± 28**
eRVSP,mmHg	38± 14	37 ± 14	39 ± 10	26± 5**	25 ± 5**	26 ± 5**
Hemoglobin, g/dL	10.8 ± 2.1	10.4 ± 1.7	11.6 ± 2.6	13.5 ± 2.1**	13.3 ± 2.3**	13.8 ± 1.6**
Albumin, g/dL	2.8 ± 0.6	2.9 ± 0.5	2.6 ± 0.6	4.4 ± 0.2**	4.4 ± 0.2**	4.4 ± 0.1**
Creatinine, mg/dL	0.82 ± 0.18	0.84 ± 0.23	0.82 ± 0.18	0.84 ± 0.19	0.86 ± 0.21	0.80 ± 0.15
Glucose, mg/dL	86 ± 14	86 ± 12	85 ± 16	88 ± 14	85 ± 15	92 ± 11
Total cholesterol, mg/dL	177 ± 32	178 ± 36	170 ± 31	183 ± 34	183 ± 35	184 ± 31
NT-proBNP, pg/mL	288 (84–1105)	133(74–343)	1239(1015–2278)‡	144 (68–292)**	113 (67–255)**	232(109–644)**
Insulin, mIU/L	8.15 ± 6.47	9.21 ± 7.03	5.71 ± 3.90†	9.13 ± 7.81	8.95 ± 7.87	9.32 ± 7.71
C-peptide, ng/mL	1.68 ± 0.96	1.71 ± 1.04	1.61 ± 0.71	1.62 ± 1.04	1.55 ± 1.03	1.74 ± 1.05
HOMA-IR	1.77 ± 1.56	1.98 ± 1.69	1.26 ± 1.00†	2.08 ± 1.94	2.01 ± 1.96	2.17 ± 1.90
BMD, g/cm^2^	1.06 ± 0.13	1.08 ± 0.13	1.04 ± 0.15	1.09 ± 0.12*	1.10 ± 0.13**	1.08 ± 0.11
Lean body mass, kg	42.5 ± 10.3	45.4 ± 10.1	36.8 ± 8.3‡	41.5 ± 9.1**	43.5 ± 8.9**	37.0 ± 8.1
Body fat, %	26.5 ± 7.6	25.4 ± 7.3	28.9 ± 7.9	28.3 ± 7.4**	27.3 ± 7.2**	30.5 ± 7.5

Values are presented as the mean ± SD or number (%) or median (IQR).

† p <0.05 vs. MR

‡ p< 0.01 vs. MR

* p <0.05 vs. before surgery

** p< 0.01 vs. before surgery.

Paired t-test or Wilcoxon signed rank test (non-parametric). BP, blood pressure;BMI, body mass index; SNAQ, simplified nutritional assessment questionnaire; LVEF, left ventricular ejection fraction;LA, left atrial; eRVSP, estimated right ventricular systolic pressure; NT-proBNP, N-terminal pro-brain natriuretic peptide; HOMA-IR, homeostasis model assessment for insulin resistance; BMD, bone mineral density.

**Fig 1:**
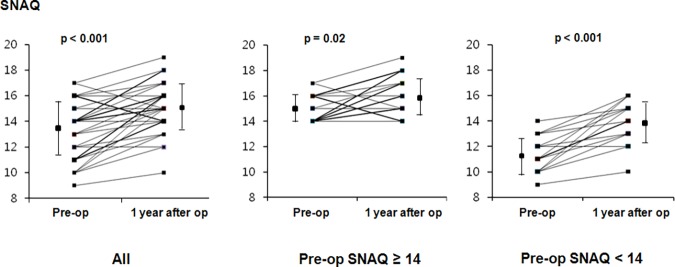
Changes in short nutrition assessment questionnaire (SNAQ) before and after mitral valvular surgery (SNAQ score <14 has been reported to be a significant risk for weight loss >5% within 6 months). Paired t-test or Wilcoxon signed rank test (non-parametric).

Despite no difference in BMI before and after MV surgery, pre-operative SNAQ for assessment of appetite was significantly increased after surgery. Patients with pre-operative SNAQ score <14, who were at a significant risk of weight loss >5% within 6 months, showed significant improvement in SNAQ score after surgery (11.1 ± 1.2 vs. 13.6 ± 1.6, p <0.001) (Fig 1).

On echocardiography, LV stroke volume was significantly increased and both LAVI and estimated RVSP (eRVSP) were significantly decreased after surgery. Low pre-operative hemoglobin and albumin levels were significantly increased and pre-operative NT-proBNP level was markedly decreased after MV surgery in both patients with MR and MS (Table 1).

On DXA for assessment of body composition, both pre-operative BMD and body fat percentage were significantly increased and pre-operative lean body mass was decreased after MV surgery in patients with MR and no changes were observed in patients with MS.

On correlation analysis of SNAQ with anthropometric, echocardiographic, laboratory data, and DXA profiles, only pre-operative eRVSP and ΔeRVSP values in patients with MR showed significant correlations with the respective values of SNAQ (Table 2).

**Table 2:** Correlation coefficients of short nutrition assessment questionnaire scores before and after mitral valve surgery according to the primary valve lesion.

Preoperative variable	SNAQ before surgery						ΔVariable	ΔSNAQ					
	All		MR		MS			All		MR		MS	
	r	p-value	r	p-value	r	p-value		r	p-value	r	p-value	r	p-value
BMI	0.140	0.366	-0.076	0.702	0.373	0.155	ΔBMI	0.286	0.107	0.488	0.025	-0.047	0.885
LVEF	0.140	0.364	0.001	0.996	-0.003	0.991	ΔLVEF	-0.142	0.376	-0.187	0.350	-0.453	0.104
Stroke volume	0.173	0.292	-0.088	0.675	0.303	0.292	ΔStroke volume	0.074	0.677	0.219	0.316	-0.304	0.364
LAVI	-0.190	0.223	-0.081	0.681	-0.024	0.934	ΔLAVI	0.162	0.317	0.412	0.033	-0.199	0.513
eRVSP	-0.308	0.047	-0.424	0.028	-0.050	0.860	ΔeRVSP	-0.295	0.068	-0.548	0.004	0.279	0.355
Hemoglobin	-0.128	0.401	-0.145	0.454	-0.112	0.680	ΔHemoglobin	0.066	0.677	0.055	0.780	-0.003	0.991
Albumin	-0.137	0.369	-0.098	0.612	-0.197	0.464	ΔAlbumin	-0.048	0.767	-0.053	0.792	0.151	0.606
Log NT-proBNP	-0.302	0.046	-0.251	0.198	0.107	0.693	ΔLog NT-proBNP	0.140	0.616	0.363	0.068	-0.430	0.125
HOMA-IR	0.120	0.445	-0.023	0.907	0.416	0.123	ΔHOMA-IR	0.016	0.924	0.134	0.523	0.122	0.679
BMD	0.143	0.348	0.115	0.560	0.067	0.806	ΔBMD	-0.067	0.676	-0.167	0.406	0.005	0.988
Body fat	0.026	0.866	-0.136	0.483	0.011	0.969	ΔBody fat	0.291	0.065	0.388	0.041	0.149	0.627
Lean body mass	0.187	0.219	0.166	0.399	0.431	0.096	ΔLean body mass	0.104	0.516	0.144	0.472	0.016	0.957

Pearsons’s coefficient or Spearman correlation. SNAQ, simplified nutritional assessment questionnaire; MR, mitral regurgitation; MS, mitral stenosis; BMI, body mass index; LVEF, left ventricular ejection fraction; LAVI, left atrial volume index; eRVSP, estimated right ventricular systolic pressure; NT-proBNP, N-terminal pro-brain natriuretic peptide; HOMA-IR, homeostasis model assessment for insulin resistance; BMD, bone mineral density.

When the patients were divided into subgroups according to the changes in body fat percentage (median value of Δfat = 2%, Table 3), majority of the anthropometric, echocardiographic, and laboratory data of both patients with MR and MS showed similar improvement after MV surgery. However, only in patients with MR, patients with Δfat ≥2% showed a significant difference in ΔeRVSP after surgery compared with patients with Δfat<2%. However, patients with MS showed no significant changes in the parameters between the patients with Δfat ≥2% or < 2%.

**Table 3:** Comparisons of changes in anthropometric, echocardiographic, and laboratory data before and after mitral valvular surgery according to the primary valve lesion with respect to the change in body fat percentage.

MR							
Variable	ΔFat< 2%			ΔFat≥2%			*p-value
	Before surgery	1 year after surgery	p-value	Before surgery	1 year after surgery	p-value	
BMI, kg/m^2^	23.3 ± 2.5	22.3 ± 2.99	0.022	23.3±3.7	24.3±3.5	0.009	0.001
SNAQ	14 ± 2	15 ± 2	0.023	13.5±1.9	15.6±1.4	0.005	0.212
LVEF, %	67 ± 4	63 ± 5	0.012	68±6	62±10	0.018	0.629
Stroke volume, ml	58 ± 12	67±12	0.139	61±13	71±11	0.011	1.000
LAVI, ml/m^2^	63±29	40±24	0.001	69±49	41±34	<0.001	0.357
eRVSP, mmHg	31 ± 8	25 ±5	0.017	41±17	26 ±5	<0.001	0.043
Hemoglobin, g/dL	10.2±1.4	13.1±2.5	0.007	10.5±1.9	13.5±2.1	0.001	0.864
Albumin, g/dL	2.9±0.5	4.3±0.2	0.001	2.8±0.5	4.5±0.3	<0.001	0.31
Total cholesterol, mg/dL	162±27	172±38	0.551	191±34	192±32	0.705	0.879
NT-proBNP, mg/dL	105 (64–207)	107 (68–211)	0.600	275 (72–374)	113 (39–311)	0.163	0.161
HOMA-IR	1.5±0.5	1.4±1.2	0.133	2.2±2.1	2.5±2.3	0.326	0.059
BMD, g/cm^2^	1.05±0.08	1.07±0.09	0.026	1.10±0.15	1.12±0.15	0.093	0.45
Body fat, %	26.4±6.8	25.0±6.6	0.033	24.6±7.8	29.1±7.3	<0.001	<0.001
Lean body mass, kg	44.9±9.3	43.4±8.2	0.245	45.9±10.9	43.6±9.7	0.002	0.578
	MS						
Variable	ΔFat<2%			ΔFat≥2%			*p-value
	Before surgery	1 year after surgery	p-value	Before surgery	1 year after surgery	p-value	
BMI, kg/m^2^	22.2±2.4	20.9±2.5	0.465	21.1±2.3	21.7±2.2	0.401	0.396
SNAQ	13.3±2.3	14.8±2.1	0.14	12.7±2.4	14.1±1.9	0.073	0.843
LVEF, %	61±3	61±6	1.000	55±14	64±3	0.068	0.120
Stroke volume, ml	48±11	56±3	0.465	49±10	65±12	0.043	0.345
LAVI, ml/m^2^	79±24	41±10	0.028	104±46	57±27	0.018	0.568
eRVSP, mmHg	32±10	24±4	0.068	44 ±10	28 ±7	0.012	0.079
Hemoglobin, g/dL	12.8±2.1	14.8±1.7	0.075	11.2±3.0	13.2±3.0	0.035	0.948
Albumin, g/dL	2.7±0.3	4.4±0.1	0.027	2.6±0.8	4.4±0.1	0.017	0.194
Total cholesterol, mg/dL	174±23	197±15	0.042	175±31	174±37	0.484	0.053
NT-proBNP, mg/dL	1054 (573–2503)	109 (18–369)	0.028	1239 (1084–2271)	285(220–809)	0.017	0.606
HOMA-IR	1.56±1.47	2.13±2.27	0.753	1.15±0.52	1.66±0.83	0.05	0.439
BMD, g/cm^2^	1.11±0.09	1.13±0.09	0.115	1.06±0.13	1.05±0.12	0.400	0.070
Body fat, %	30±10	28±8.8	0.173	27±6	32±6	0.012	0.003
Lean body mass, kg	39.2±10.7	38.7±10.1	0.345	37.0±6.5	35.7±6.6	0.208	0.302

*Comparisons of Δ values between the subgroups. Values are presented as the mean ± SD or median (interquartile range). Wilcoxon signed rank test for paired variables or Man-Whitney U test for independent variables. MR, mitral regurgitation; MS, mitral stenosis; other abbreviations as shown in Table 2.

**Fig 2:**
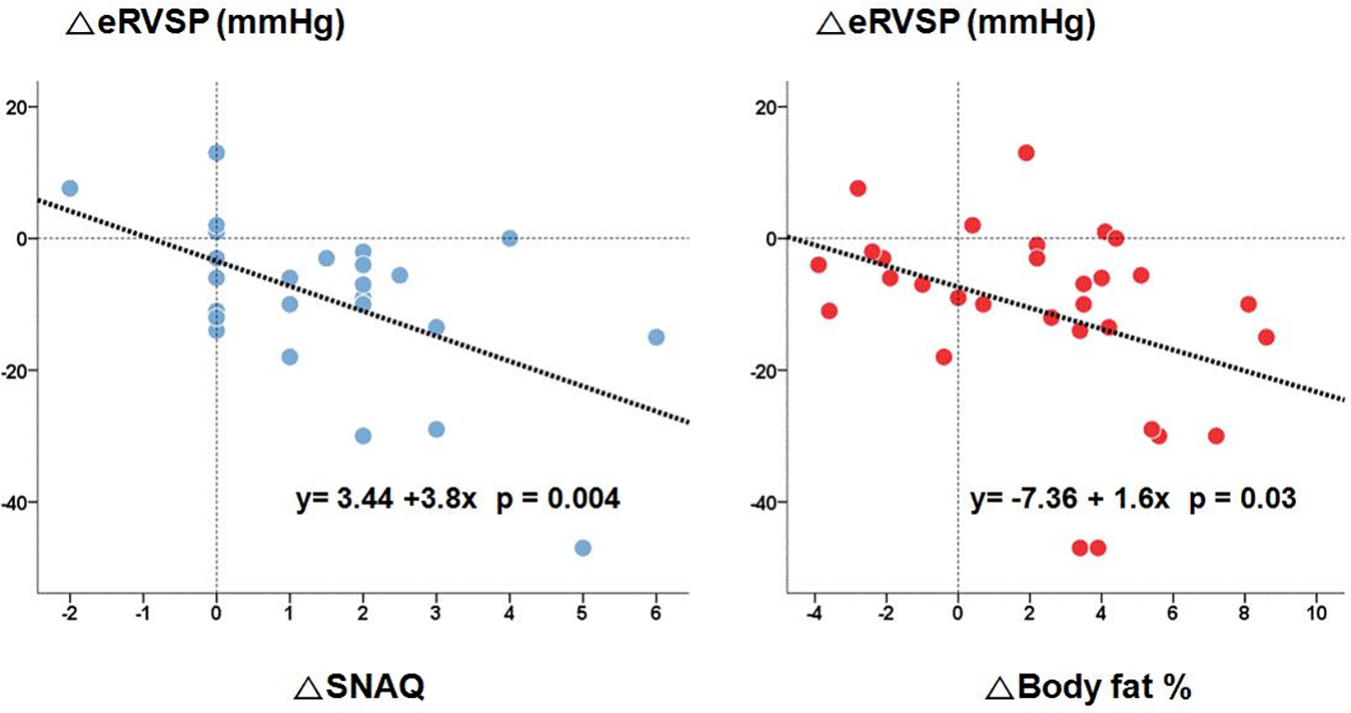
Relationship of ΔSNAQ and body fat percentage with ΔeRVSP in patients with mitral regurgitation. Pearson’s coefficient(r). SNAQ, short nutritional assessmentquestionnaire; eRVSP, estimated right ventricular systolic pressure.

While analyzing the relation with ΔeRVSP, both ΔSNAQ and Δfat% had an inverse correlation with ΔeRVSP (p = 0.004 and p = 0.03, respectively) (Fig 2).

## Discussion

The present study demonstrated that patients with severe MV disease showed an improvement in their appetite, laboratory data as well as hemodynamic parameters after MV surgery.

In respect to body composition, although body weight did not change after surgery, significant increase in body fat percentage and BMD were observed in patients with MR. Additionally, the changes in SNAQ and body fat percentage showed close relations with the magnitude of change in eRVSP in those patients with MR. These findings suggest that corrective MV surgery brings favorable outcome in wasting process as well as hemodynamic improvement, and that RV pressure overload is significantly associated with the changes in appetite and body composition, especially in patients with severe MR.

### Body alterations after MV surgery

The alteration of body composition in wasting process consists of a reduction in fat mass, fat-free mass and bone mass [4–5]. Palmieri V. et al. suggested that more severe valvular insufficiency is associated with lower body mass index (BMI) and lower body fat mass, measured with bioimpedance [11]. Studies in dogs with mitral stenosis showed that cachectic changes became more pronounced and they lose fat and lean body mass over time [12].

For the reversibility of wasting process, patients with heart failure who underwent heart transplantation, showed an improved cardiac function leading to the cessation of wasting process with subsequent weight gain[13]. However, weight gain following the transplantation is also associated with the use of steroid and immune suppressants that cause increase in fat mass [14].

To our knowledge, this is the first study to demonstrate the reversibility of wasting process with the changes in body composition after corrective surgery for valvular heart disease. Majority of our patients with severe MV disease, who were scheduled for MV surgery, showed normal range of BMI with non-cachectic appearance preoperatively. As healthcare accessibility and HF management including cardiovascular medications improve, patients with typical cachectic appearance are hardly seen in recent practice of cardiology.

Nonetheless, our population showed evidences of significant malnourishment, as indicated by anemia, low albumin level, as well as poor DXA profile (low body fat percentage and BMD).

After MV surgery, the body profile alterations in our population could be contributed to a certain extent by the significant increase in their appetite after surgery. Similarly, Buchanan N. et al. reported that 11 patients with severe MV disease had experienced a marked increase in appetite after MV surgery [6].Though the precise mechanism is unclear, neurohormonal activation involved in poor appetite in HF might be alleviated by the corrective MV surgery, which improves the cardiac function. In laboratory findings, NT-proBNP, a surrogate of high LV filling pressure that stimulates lipolysis in adipose tissue [15], significantly declined and serum albumin increased after surgery. It identifies a nutritional improvement in patients recovering from heart failure after corrective MV surgery.

Furthermore, body compositionof this population showed alteration as an increase in body fat percentage and BMD, despite no significant difference in the BMI before and after MV surgery. As wasting process progressed in HF, fat loss precedes the loss of lean body mass, and alterations in lean body mass may be either inconclusive or decreased [16]. Melenovsky V. et al. had identified the decrease in fat mass and relative preservation of lean body mass in cachectic patients with advanced HF by using DXA [17]. In these patients with MR, although the depleted body fat and BMD were replenished over one year, lean body mass was significantly decreased after surgery. Considering that lean body mass detected by DXA is heterogeneous and consists of cell mass and total body water distributed in intracellular and extracellular spaces, the significant decrease of lean body mass In this study may reflect the loss of edema (body water) rather than the loss of cell mass with an improvement of heart failure after corrective surgery.

When we dichotomized the patients according to the primary valve lesion, both groups showed an improvement in appetite, echocardiographic and laboratory parameter in the same manner. Notably, even patients with MS were undersized, they had higher percentage of body fat than patients with MR, preoperatively. After surgery, only patients with MR showed a significant increase in BMD and body fat and the patients with MS did not. It suggests that body wasting in pre-operative patients with MS might be less advanced and surgical correction had less impact on body composition in patients with MS. However, it should also be taken into account low statistical power by small sample size of patients with MS.

### RV impairment and body wasting

On correlation analysis, pre-operative SNAQ and ΔSNAQ were significantly associated with the respective values of eRVSP, and these results are limited to the patients with MR. Also, patients with Δfat ≥2% showed higher pre-operative eRVSP and ΔeRVSP levels than the patients with Δ fat <2% in patients with MR. This demonstrated that both ΔSNAQ and Δfat % have inverse relationships with ΔeRVSP and that RV pressure overload is involved in the occurrence and recovery of wasting process in severe MV disease, especially in MR. However, these results do not apply to the patients with MS despite the similar eRVSP values with those with MR. Among left-sided valvular heart disease, pathophysiological adaptation in MS begins with chronic pressure overload of LA and chronic pressure and volume overload in MR. By non-compliant LA, pulmonary venous pressure is elevated and lead to irreversible changes as deposition of type II collagen, which passively increases pulmonary arterial pressure (PAP). The difference in the two diseases is that PAP in MS is rapidly decreased after relief of the stenosis, whereas more time could be required in MR of which pulmonary hypertension is linked to volume overload [18]. These different responses of PAP after MV surgery might be involved in the different results of the two diseases in this study, although the precise mechanism remains unclear. However, as mentioned above, we should also consider the sample size and less advanced body wasting of patients with MS than those with MR. Further studies with more patients with valvular heart disease and serial follow-up are needed to confirm our results.

In this study, we found no significant relationship between the changes in LV systolic function and body composition, which is line with the previous reports that LV EF was similar in both cachectic and non-cachectic patients [4,19].

Although there are few data about the relation between RV function and wasting process, growing evidence suggests the importance of RV function and the associated parameters as principal factors of wasting process in HF [17, 20–22]. It has been reported that increased right atrial pressure and tricuspid regurgitation (TR) were significant predictors of malnutrition and were associated with the loss of fat mass in HF [20–22]. Melenovsky V. et al. identified that patients with RV dysfunction had lower BMI and less body fat mass than those with preserved RV function, whereas lean body mass was similar in both groups [17].

For the pathogenesis of wasting process, RV impairment leads to splanchnic venous congestion, liver stasis, and gastrointestinal edema, and it might be associated with anorexia, protein, and fat malabsorption. The catabolic effect of inflammatory cytokines and neurohormone might also play an important role in weight loss [23]. However, the underlying pathogenesis beginning with RV impairment until wasting is not fully elucidated.

The reversing of wasting process with improvement of RV dysfunction has been reported earlier, albeit small studies. Serum protein level in a patient with severe TR complicated by severe hypoproteinemia became normalized after tricuspid valve replacement [24], and patients with atrial septal defect (ASD) and massive TR showed a significant weight gain after successful ASD device closure [25]. Patients with HF and severe pulmonary hypertension, who underwent phosphodiesterase 5 inhibitor therapy, showed a significant decrease in pulmonary arterial pressure and maintained a steady body weight when compared with controls who experienced significant weight loss [26].

In this study, the patients with severe TR and RV dysfunction were excluded to assess the isolated effect of severe MV disease on body composition. Nonetheless, we found that RV pressure overload in itself could affect the body composition in severe MV disease and these results support the previous studies suggesting the crucial role of RV in the wasting process of HF.

### Limitation

There are several limitations in this study. First, the study population was relatively small; a larger sample size and serial follow-up would provide more conclusive results. Although the noninvasive estimation of RVSP using echocardiography has been validated with invasively measured pressures in numerous studies, right heart catheterization would be the definitive modality for assessment of right heart pressures and function. To assess isolated effect of severe MV disease on body composition, we excluded the patients with low LV systolic function and other cardiomyopathies who would be expected to gain greater changes to the wasting process. Also, patients with RV dysfunction and severe TR were excluded and these results are restricted to the patients of MV disease without overt RV dysfunction. Since the corrective surgery in isolated TR and RV dysfunction cases are limited, it is hard to determine the role of RV dysfunction on wasting process. Most of patients had taken cardiovascular medications as statin, beta blocker and/or calcium channel blocker, which could also affect on the metabolism. Since it is difficult to consider all of these aspects, we exclude only patients taking medications which directly influence on the appetite as steroid, anti-depressant, and other appetite stimulants.

## Conclusions

We demonstrated that corrective MV surgery contributed to favorable outcomes in wasting process as well as hemodynamic improvement in patients with severe MV disease. Particularly, patients with higher reduction in RV pressure after MV surgery showed greater improvement in appetite and increment in body fat percentage in patients with MR. This indicates the role of RV pressure overload on wasting process in patients with severe MV disease.

## Supporting information

S1 Dataset Dataset containing the parameters used in this study. (SAV)
